# The Humanity of Medicine

**Published:** 1894-06-09

**Authors:** 


### The Hospital

AN INSTITUTIONAL JOURNAL OF

The Medical Sciences and Hospital Administration,

WITH

AN EXTRA NURSING SUPPLEMENT.

VOL. XVI.—No. 402.] [JUNE 9, 1894.

### THE HUMANITY OF MEDICINE.

No profession makes smaller pretensions to humanity than that of medicine: no profession is in daily habit and practice more humane. The outward husk of the modern doctor of the best class is often scientifically hard, but every man who in these times of financial straitness can afford occasional dessert is familiar with certain nuts which have very hard shells, but which, when once you have deftly cracked the shells, are as full as they can well be of kernel of the finest quality. It is exactly so with the British doctor of the *fin de siècle*. He will not thank you for calling him humane. Mere sentimentality he distrusts, and hates with a strong hatred. But give the surgeon a man with a broken limb, or the physician a child frantic with pain and fever, and you behold at once the spirit of modern humanity in its noblest, tenderest, most patient, and strongest form. What the doctor cannot put into poetry or prose he can put into action. He is the Good Samaritan of the nineteenth century multiplied a thousand-fold, and distributed over every street and lane of country and town.

With most men and callings the primary object is the money which can be made. This is not said as a reproach. The making of money is a perfectly legitimate, and indeed an indispensable object in life. It is probable that the majority of doctors when they first decide upon medicine as a profession choose it because they consider that it will enable them to live comfortably as educated persons. But this result almost invariably follows with men of character who practice the medical calling for a number of years, that the healing work of the profession becomes to them infinitely more important than the commercial rewards. It is rare to see a doctor who is a mere money-lover; it is almost rarer still to find one who is not alive from head to foot with the instincts of the highest humanity in the presence of sickness or pain. We are not saying these things in praise of individual doctors, but in praise of the calling of medicine. In itself medicine is so humane, and modern medicine is so severely scientific and honest as well as humane, that the mere practice of it transmutes ordinary bronze humanity into fine gold.

Should modern medicine be encouraged or discouraged? This is a question which ordinary people seldom ask themselves or one another. Medicine, they think, must fight its own battles, as the science of engineering or the art of tailoring is compelled to do. But this attitnde of mind does not quite fulfil the ideal which the spirit of the times sets before us. The "advanced" politician professes that his aim is to elevate humanity in material well-being and mental content. But there are some ideas, some general habits, some arts, some callings, which make greatly for the general happiness and well-being; and there are others which make in an opposite direction, or which at least have a merely neutral value from the point of view of public well-being and happiness. What of medicine when tried by this standard? What of surgery? What of skilled nursing? What of anæsthetics and general sanitation? Modern medicine, surgery, nursing, and sanitation are victorious achievements over ignorance, helplessness, painfulness, and death in a thousand melancholy, pitiful, and even hateful forms. No one loves pain, or to have his work interrupted by prolonged illness, or his career cut short by some ridiculous bacillary accident, such as defective drainage may occasion. It is humiliating to a high-spirited man or race of men to be at the mercy of creatures and circumstances which, so far as we know, have neither intellect nor purpose even in their lowest and most rudimentary forms. Modern medicine puts forth her hand and plucks us away from this reign of ignorance, helplessness, and fortuitous, though deadly, accident. She gives us knowledge, and sets before us certain narrow ways of health and safety, which in most instances will bring us to the solid rewards of enjoyable health and a completed career.

These are the aims of medicine. No one can deny that they are great aims. From the point of view of medicine itself they are unselfish aims. The doctor does not acquire his knowledge for himself. So far from that, we find him constantly plunging himself, with his eyes open, into the most instant and deadly dangers in the daily exercise of his calling. Of the nurse this may be said with at least as much, and almost more of general application. The aim of the doctor is, first, the cure of his individual patient; secondly, the scientific advancement of his art; and thirdly, the perfect health and happiness of the community. This we hold to be a very great series of aims indeed; well worthy of the energies of a separate calling or profession, and well worthy of the devotion of the best intellects of the time. Moreover, for advanced civilisation, consisting as it does in the massing together of enormous populations at a few centres, and the weakening of the ordinary forces which make for nerve, energy, and happiness, such as a pure atmosphere and an almost universal open-air life, we hold a scientific and practical medicine to be quite indispensable.

Now there is a very obvious conclusion from all this. The conclusion is that one of the most effective instruments of progress and happiness in an advanced civilisation is scientific medicine. The public, for whom this medicine exists, will show exactly the wisdom of the soldier who does not take the trouble to "keep his powder dry" if it does not take whatever trouble is required to keep its medicine and its medical practitioners thoroughly bright and up to date in all the details of their science and art. There are a good many ways in which this can be done, of which we shall be glad to speak at fitting seasons. At present we wish to point out one way. The hospitals have this peculiar feature, that they are both practitioners of medicine and makers of doctors. If then the public is wise enough to resolve that it will secure for itself the invaluable aid of a thoroughly scientific medicine in its struggles with modern difficulties, it will, in the first instance, make straight for the hospitals. It is there that modern medicine takes its rise; and as is the source such must be the rivers and streams which flow from it.

In order that the hospitals may be extensively successful in raising humanity to a higher level of practical happiness and efficiency two things are required at the hands of that part of the public which reasons: first a constant and candid interest in hospital politics; and secondly, a generous and unremitting material support. We do not make our appeal to the mere self-interest of the community when we ask that it shall study hospital politics and charge itself with the maintenance of hospitals. Our appeal is to individuals to preserve and improve for humanity's sake a set of institutions which together constitute, as we have shown, one of the most efficient of all known instruments of human happiness and progress. Hospitals are to be maintained and constantly improved for the sake of what they can do for the whole community and every class in it. They must be supported on precisely the same grounds as those on which we base our appeals for the support of education or religion. Perhaps at the present moment a more special appeal will be held to be not only justifiable but welcome. We do not desire to *worry* men and women into giving. We know they are willing and anxious to give. But we are desirous that they shall be well informed; that they shall feel themselves justified, not only in giving, but even in denying themselves at a time of straitness for the sake of giving as much as possible. The justification for special efforts at the present moment is that hospitals are feeling the pressure of the times in a marked degree in every department of their activity, and that they are institutions which are so imperatively necessary for the daily relief of the sufferers amongst us, and so indispensable to the continued well-being, progress, and happiness of the whole community, that however much is done for them, and at whatever sacrifice, it can never be unnecessary and never unwise. To keep the hospitals in a condition of first rate activity is on a par with keeping our navy strong, our army ready, our schools efficient, and our churches full of life.

